# Assessment of the Levels of Potentially Toxic Elements Contained in Natural Bentonites Collected from Quarries in Turkey

**DOI:** 10.1021/acsomega.3c01773

**Published:** 2023-05-30

**Authors:** Aydan Altıkulaç, Şeref Turhan

**Affiliations:** † Ula Ali Koçman Vocational School, Muğla Sıtkı Koçman University, Ula, 48640 Muğla, Turkey; ‡ Department of Physics, Faculty of Science, Kastamonu University, 37150 Kastamonu, Turkey

## Abstract

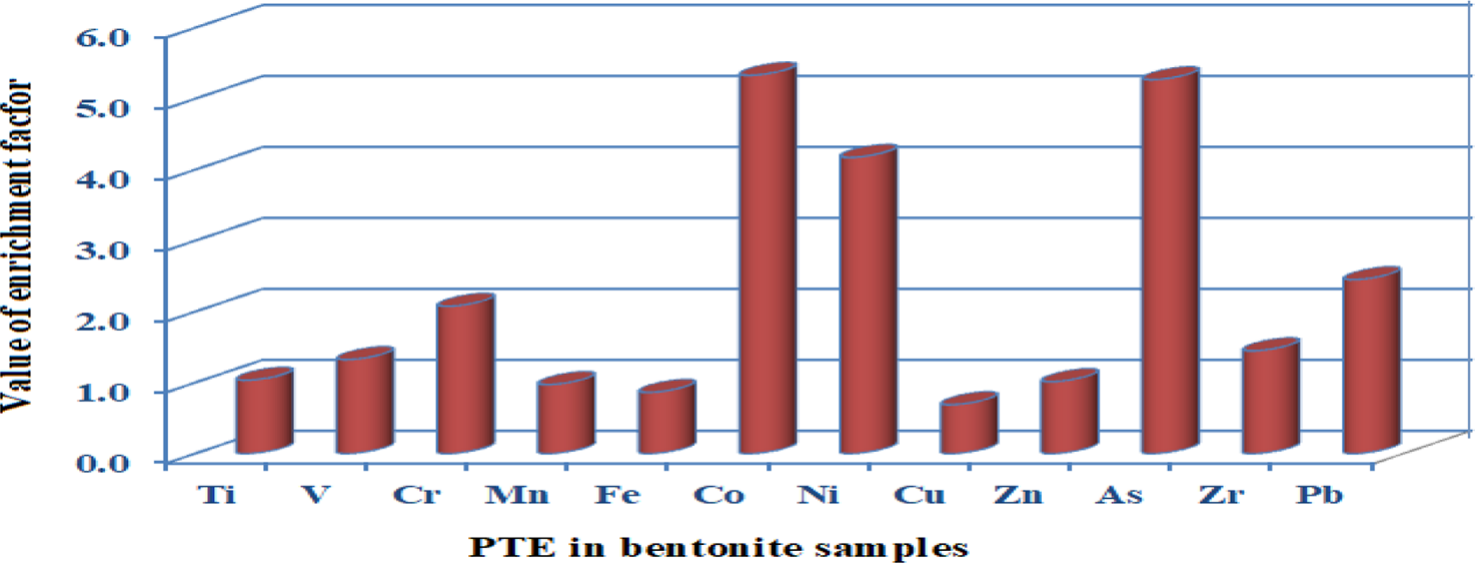

Potentially toxic elements (PTEs) are an important type of pollutant, causing constant and far-reaching concerns around the world due to their increase in the mining process. Bentonite formed by the alteration of glass-rich volcanic rocks is a smectite clay consisting mostly of montmorillonite. Bentonite is an important mineral used in a wide range of applications in many fields such as oil and gas, agriculture, food, pharmacological, cosmetic, and construction industries due to its unique qualities. Given the widespread distribution of bentonite in nature and its use in a wide variety of consumer products, it is inevitable that the general population will be exposed to PTEs contained in bentonites. In this study, concentrations of PTEs in 69 bentonite samples collected from quarries located in different geographical regions of Turkey were analyzed by an energy-dispersive X-ray fluorescence spectrometric method. The average concentrations of Ti, V, Cr, Mn, Fe, Co, Ni, Cu, Zn, As, Zr, and Pb in bentonite samples were found to be 3510, 95, 129, 741, 30,569, 67, 168, 25, 62, 9, 173, and 28 mg/kg dry weight, respectively. Results of the enrichment factor relating to Earth’s crust average indicated moderate enrichment with Cr, Ni, and Pb and significant enrichment with Co and As.

## 1 Introduction

Recently, environmental pollution, which adversely affects humans, animals, plants, and ecosystems, has become a serious and important problem throughout the world due to rapidly increasing population growth, accelerated urbanization, and industrial developments.^1−3^ Potentially toxic elements (PTEs, heavy metals or metalloids) are an important pollutant since they are persistent and non-degradable.^1,4^ In PTE analyses of environmental samples (soil, water, sediment, etc.), lead (Pb), mercury (Hg), arsenic (As), cadmium (Cd), chromium (Cr), cobalt (Co), and nickel (Ni) are of great importance due to their high toxicity and potential risks to both human health and urban ecosystems.^5^ These PTEs affect the central nervous system and disrupt the normal functioning of internal organs.^6^ They are cofactors in the development of cardiovascular and respiratory diseases. Other PTEs such as manganese (Mn), iron (Fe), zinc (Zn), and copper (Cu) are essential for human metabolism, but high concentrations of these elements can have detrimental effects on human health.^2^ PTEs in the environment originate from various human activities (mining, chemical, and metallurgical industries, smelting procedures, agriculture, traffic, etc.) and natural (lithogenic) sources such as volcanic eruptions and weathering of element-containing rocks.^4,5^ Mining activities involving many processing methods such as grinding the rock and ores, recovering the desired fraction, and dumping the waste into a tailing or holding pond are among the main sources of PTEs in the environment.^7−9^ PTE elements released into the surrounding environment during mining not only affect soil quality but also threaten food safety of crops grown in polluted soil and human health. In addition, exposure to PTEs can pose a health problem for miners.^7,9−13^

Bentonite mineral is formed by devitrification and the accompanying chemical alterations of pyroclastics and/or volcanoclastic rocks.^14,15^ It consists of montmorillonite, which is one of the smectite groups of clay minerals.^14^ Bentonite mineral is processed to obtain sodium and calcium montmorillonite, active clays, and organo-clays.^16^ However, from a commercial point of view, there are two primary types of natural bentonite: calcium (Ca) bentonite (or non-swelling bentonite) and sodium (Na) bentonite (or swelling bentonite).^1^ Natural and modified bentonites are used either directly or as industrial raw materials in a wide range of applications such as drilling muds, pet litters, waterproofing and sealing applications, animal feed additives, oil and grease absorbents, agricultural carriers, filtration, clarification, decolorizing agents, asphalt emulsions, catalysts, and additives in the food, cosmetic, pharmacological, and construction industries, and so forth depending on their physical and chemical properties.^17^ There are considerable reserves of bentonite (approximately 370 million tons) in Turkey, distributed in different geographical regions, especially in Central Anatolia and the Black Sea Region.^7^ With an annual production of 1.5 million tons, Turkey is the fifth highest bentonite-producing country after the USA, China, India, and Greece.^7^ In Turkey, bentonites are generally used as drilling mud, binder (foundry-sand bond and iron ore pelletizer), and cat litter, while most of the bentonites produced are exported to European Union countries.^7^

Bentonite is usually exploited in open quarries by surface mining.^18^ The bentonite production process involves ore mining, and if necessary, sodium activation by adding soda ash (Na_2_CO_3_) to convert Ca bentonite to Na bentonite, drying to obtain the required moisture content, and grinding.^16^ Moreover, bentonite mining generates a large amount of waste that has a significant environmental impact and has no commercial value. Because the particles are so fine that they can be transported through the air and can penetrate and settle in the lungs, improper waste disposal causes soil and groundwater pollution as well as poses risks to fauna, flora, and human health.^19^ Considering the widespread distribution of bentonite in nature and its use in a wide variety of consumer products, it is inevitable that quarry workers, the general population, and the environment will be exposed to PTEs contained in bentonite. From this point of view, it is important to know the concentrations of PTEs contained in bentonites. Until now, many studies on the absorption/adsorption and desorption of PTEs or heavy metals by bentonites have been published in the literature.^20−33^ However, according to our literature research, there is no detailed study on the determination of PTE concentrations in bentonites. This study aims to determine the concentrations of PTEs (Ti, V, Cr, Mn, Fe, Co, Ni, Cu, Zn, As, Zr, and Pb) in 72 bentonite samples collected from quarries located in different geographical regions of Turkey using energy-dispersive X-ray fluorescence (XRF) spectrometry and calculate the enrichment factor to Earth’s crustal average of the PTEs. So, this study represents the first attempt to raise awareness for bentonite consumers and mine workers about the presence of PTEs accompanying Turkish bentonites and to establish a database of distributions of PTEs in bentonite quarries (BQs).

## 2 Materials and Methods

### 2.1 Sample Collection, Handling, and Preparation

**Figure 1:**
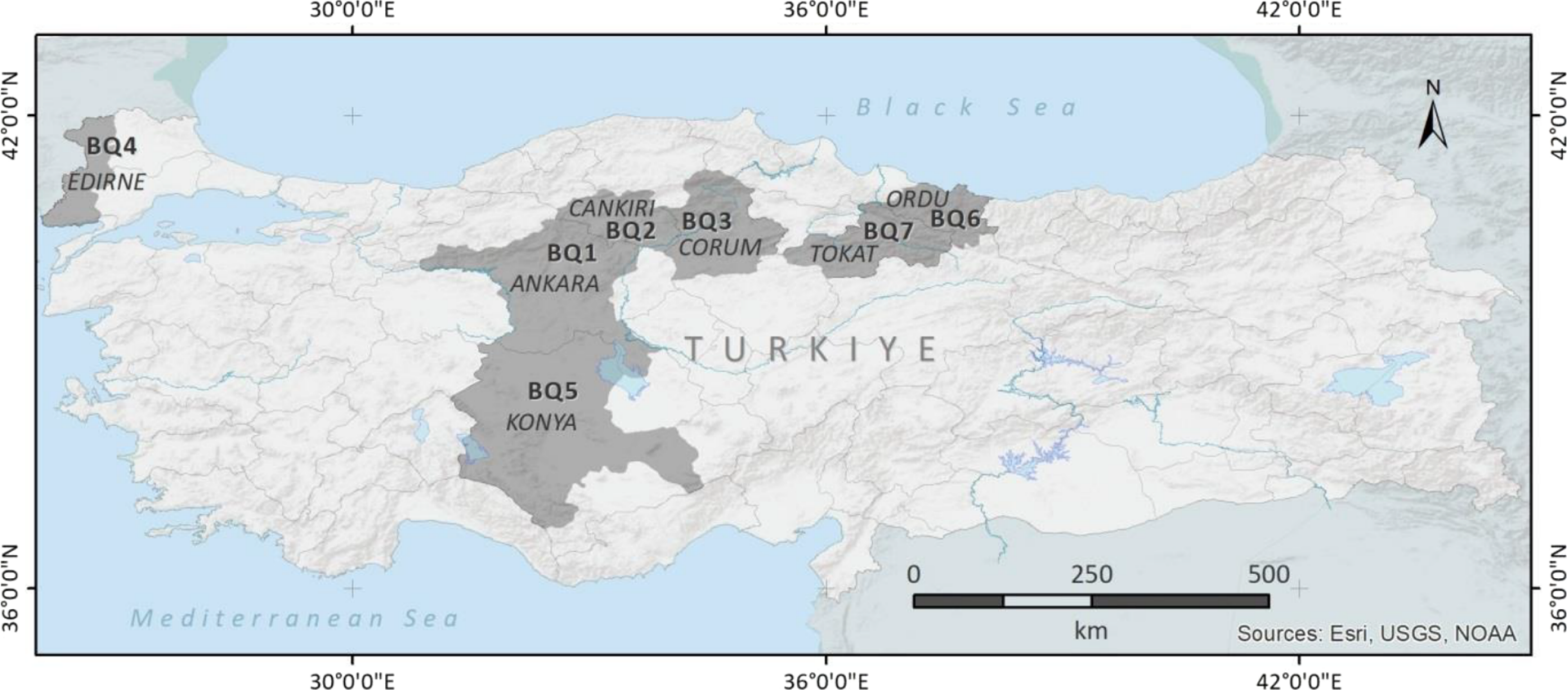
Locations of bentonite quarries.

Most of the bentonite deposits in Turkey are formed as a result of the decomposition of volcanic tuff or ash material stored in marine or lacustrine environments.^14^ The Ca bentonite deposits in Turkey are more than the Na bentonite deposits. Ca bentonite deposits are located in Balıkesir, Edirne, Ordu, Giresun, and Konya provinces, while Na bentonite deposits are located in Ankara, Çankırı, Çorum, and Tokat provinces.^14^ In total, 69 natural bentonite samples were collected from BQs located in Ankara (BQ1), Çankırı (BQ2), Çorum (BQ3), Edirne (BQ4), Konya (BQ5), Ordu (BQ6), and Tokat (BQ7) provinces of Turkey, as shown in Fig. 1.^7,14^ Bentonite samples were taken from the upper layers of each quarry, that is, from a depth of 0–5 cm. Each bentonite sample placed in polyethylene bags was brought to the sample preparation laboratory. After the samples were kept in the open air in the laboratory for a while, they were dried in a furnace at 110 °C for 5–10 h to remove moisture.^7^ The dry samples were grounded and powdered to make them fit the calibrated powder geometry in the energy-dispersive XRF (EDXRF) spectrometer.^7,34^ Each powder sample was homogenized with an agate pestle and made ready for elemental analysis.^34^

### 2.2 PTE Analysis in Bentonite Samples

Non-destructive methods such as neutron activation analysis, charged particle activation analysis, XRF, and particle-induced X-ray emission are fast, accurate, precise, sensitive, and reliable analysis techniques capable of performing simultaneous multi-element determinations.^35^ The X-ray emission underlying the XRF technique is simple, systematic, and relatively independent of the chemical state and has uniform excitation and absorption based on an atomic number. Interference in the X-ray peak in the spectrum can be easily corrected, thus ensuring high accuracy and sensitivity easily. EDXRF and wavelength-dispersive XRF spectroscopy are used for qualitative and quantitative multi-element analysis of major, minor, and trace elements in archeological, geological, biological, industrial, food, and environmental samples and require minimal sample preparation.^2,36−44^ The analysis of PTEs in the bentonite samples was performed with the help of the EDXRF spectrometer (Spectro Xepos, Ametek). The EDXRF spectrometer is equipped with an anode X-ray tube (50 W, 60 kV) consisting of a dual thick Pd/Co mixture.^7,34,37,41,43^ The EDXRF spectrometer’s target modifier, which optimizes excitation by using polarization and secondary targets, has many different excitation conditions that guarantee the best detection of all elements from Na to U. Analyses are performed in the He gas environment. The spectral resolution of the system is lower than 155 eV. The EDXRF spectrometer has 12 automatic sampling devices and software to analyze samples at the same time. It uses sophisticated calibration techniques such as “no-standard” calibration, often based on the basic parameters method. Soil-certified reference material (NIST SRM 2709) was used for quality assurance of the EDXRF system.^7,34,37,41,43^ Sample containers prepared for each bentonite sample were placed in an automatic sampler, and the analysis procedures were completed by counting for 2 h. The total uncertainty of the analytical procedure is between 2 and 15%. The XRF spectrum of each bentonite sample obtained was evaluated with the help of the software installed in the system.

### 2.3 Enrichment Factor

The enrichment factor (EF) is an effective normalization tool widely used to separate PTEs of natural variability from element fractions associated with anthropogenic activities.^4,37,45^ In this study, the EF to Earth’s crustal average was used to evaluate the degree of PTEs in the bentonite samples. EFs for Ti, V, Cr, Mn, Fe, Co, Ni, Cu, Zn, As, Zr, and Pb in the bentonite samples in all sampling areas were calculated as follows:^34,37^
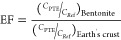
1where *C*_PTE_ and *C*_Ref_ are the concentration of PTEs and reference element in the bentonite sample and Earth’s crust, respectively. When the relevant literature is examined, it is seen that elements such as Al, Mn, Fe, Ca, Zr, Sc, Sr, and Ti are used as references based on different reasons in the calculation of the EF.^37,46−48^ In this study, Sr was used as a reference element due to its low occurrence. Sr is also one of the main components of the earth’s crust, and its concentration in the soil is also associated with some matrices. The Sr concentration in each bentonite sample was measured by using the EDXRF spectrometer. The EF values consist of five classifications as given in Table 1.

**Table 1:** Values of Enrichment Factor and Enrichment Levels

EF < 2	deficient to minimal enrichment
2 ≤ EF < 5	moderate enrichment
5 ≤ EF < 20	significant enrichment
20 ≤ EF < 40	very high enrichment
EF ≥ 40	extreme enrichment

## 3 Results and Discussion

**Figure 2:**
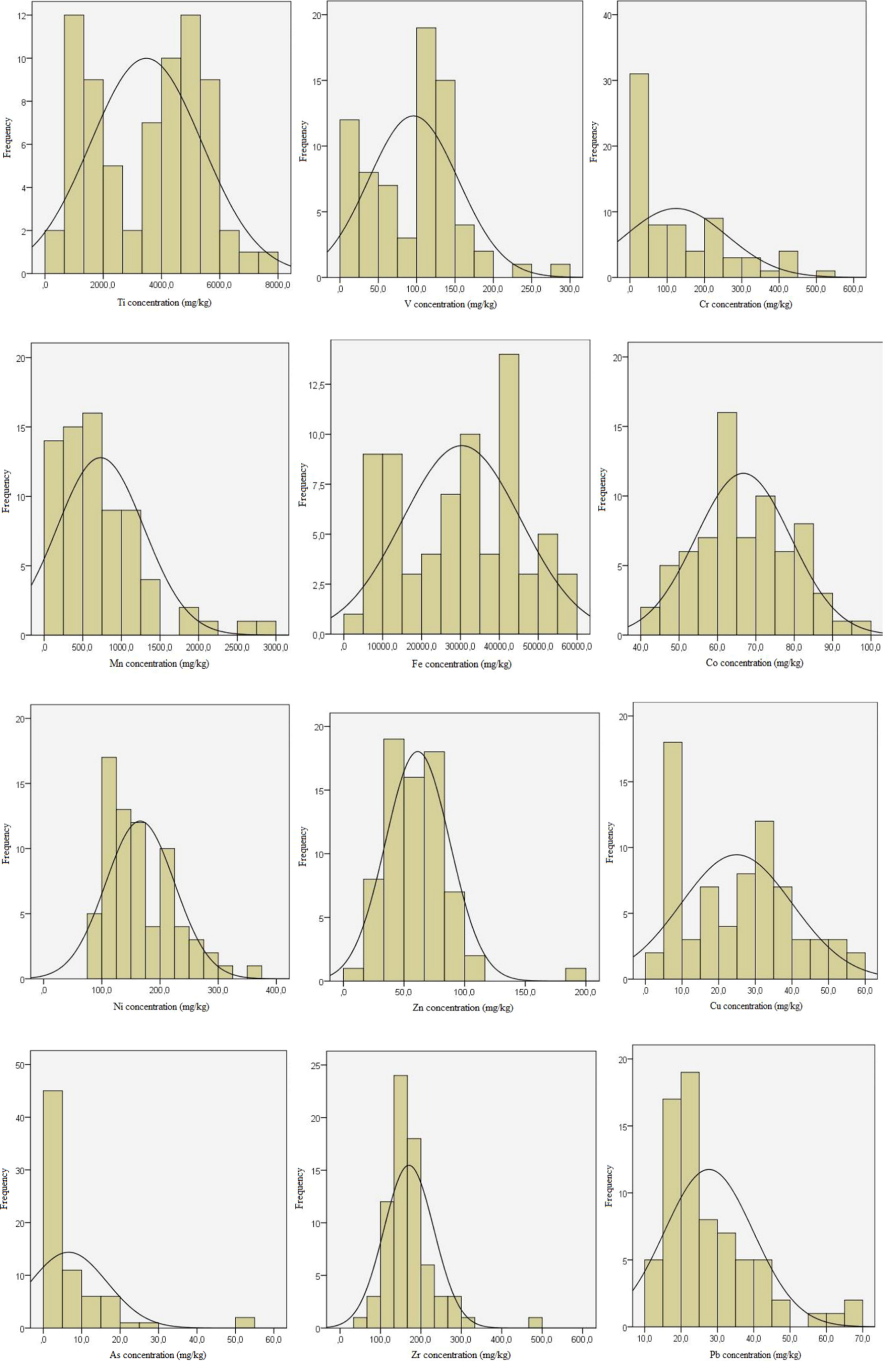
Frequency distribution of PTE concentrations analyzed in all bentonite samples.

Some descriptive statistical data related to the concentrations of PTEs analyzed in all bentonite samples and PTE concentration distributions in BQs are presented in Tables 2 and 3, respectively. The frequency distribution of the concentration of PTEs is shown in Figure 2. The average values of EF calculated for PTEs analyzed in each quarry and all bentonite samples are given in Table 4. It can be seen from Tables 2 and 3 that the concentrations of the PTEs in bentonite samples vary depending on the geological and geochemical structure of the location of the quarries. The average concentrations of the PTEs (mg/kg) were in the following order: Fe (30568.9) > Ti (3510.4) > Mn (740.8) > Zr (172.5) > Ni (168.1) > Cr (128.5) > V (94.7) > Co (66.9) > Zn (62) > Pb (27.9) > Cu (25.1) > As (9.3). The concentrations of Cd and Hg, which are very toxic elements, were found to be below the detection limit of 2.1 and 1.8 mg/kg, respectively. From Figure 2, the concentration distributions of Ti, V, Mn, Fe, Ni, and Pb in all bentonites exhibit a non-normal distribution, while Zr, Zn, Co, and Cu have a near-normal distribution. The frequency distributions of Cr and As exhibit the log-normal distribution.

**Table 2:** Some Descriptive Statistical Data of PTEs Analyzed in Bentonite Samples^a^

	the concentration of potentially toxic elements (mg/kg)											
	Ti	V	Cr	Mn	Fe	Co	Ni	Cu	Zn	As	Zr	Pb
average	3510.4	94.7	128.5	740.8	30568.9	66.9	168.1	25.1	61.8	9.3	172.5	27.9
SE	232.7	6.5	16.6	68.5	1843.7	1.5	7.2	1.8	3.2	1.6	7.4	1.5
median	3980.0	108.3	79.2	615.1	32610.0	65.4	156.2	26.6	63.0	6.2	159.5	23.9
SD	1933.0	54.0	137.5	568.9	15315.0	12.5	59.6	15.1	27.0	11.2	61.9	12.4
kurtosis	–1.2	–0.8	0.3	3.1	–1.1	–0.6	0.5	–0.9	8.3	8.4	9.3	2.2
skewness	–0.1	–0.1	1.0	1.6	–0.1	0.1	0.9	0.3	1.9	2.7	2.2	1.5
min	491.8	3.6	2.7	120.4	3968.0	43.0	79.5	3.3	14.4	0.9	40.2	10.6
max	7642.0	234.4	537.1	2833.0	59310.0	96.0	355.1	58.7	198.4	53.0	487.5	68.6

a SE: Standard error; SD: standard deviation.

**Table 3:** Average and Range (Min–Max) of Concentrations of PTEs in Bentonite Quarries

PTE		concentration (mg/kg)						
		BQ1	BQ2	BQ3	BQ4	BQ5	BQ6	BQ7
Ti	average	4697.6	5474.4	3934.9	3073.2	4195.5	912.4	2093.3
	range	2573.0–5798.0	3396.0–7642.0	2734–4805	1225–5427	3516–5183	491.8–1402	1618.0–2650.0
V	average	119.0	132.3	138.4	84.7	139.1	17.6	62.4
	range	69.6–141.4	89.1–173.5	101.4–234.4	41.1–142.3	114–175	3.6–31.6	44.7–102.9
Cr	average	194.0	283.5	156.9	36.4	84.8	6.3	6.1
	range	116.3–271.2	106.5–537.1	26.2–416.1	6.1–79.2	53.8–117.5	2.7–14.4	2.7–12.8
Mn	average	1126.6	867.0	564.3	537.3	576.0	634.7	793.2
	range	120.4–2833.0	165.1–2008.0	234–1031	154.5–1180	254.3–858	155.1–1965	215.3–1178.0
Fe	average	39371.3	42261.7	38432.2	26515.6	39142.5	9094.9	26008.3
	range	26240.0–51200.0	26990.0–59310.0	21,160–56,490	12,060–41,410	33,700–45,670	3968–12,600	18690.0–35190.0
Co	average	73.2	74.5	67.0	58.6	61.9	58.2	73.0
	range	61.1–87.8	52.1–96.0	51.4–81	44.1–80.1	50.8–73	43–84.4	55.7–85.0
Ni	average	222.5	224.0	161.7	124.6	174.2	110.7	142.0
	range	168.9–314.4	126.1–355.1	131.7–194.3	101.9–159.2	122.9–207.3	79.5–134.8	112.6–163.7
Cu	average	33.4	36.4	33.8	21.4	31.3	6.7	14.9
	range	15.4–53.3	25.1–58.7	19.1–50.3	6.2–51.9	23.4–37.0	40605.0	9.3–19.3
Zn	average	72.0	70.5	63.2	54.1	106.2	36.3	66.0
	range	46.4–102.6	40.6–97.3	42.9–104.1	33.7–82	62.7–198.4	14.4–91.4	44.9–84.7
As	average	4.6	18.2	2.8	4.1	18.5	2.3	3.0
	range	1.5–9.0	6.4–53.0	<0.8–4.9	<0.8–8.6	13.2–25.4	<0.8–5.1	<0.8–6.2
Zr	average	164.3	184.9	161.4	222.9	159.5	133.9	192.3
	range	139.6–211.0	127.4–282.2	110.5–197.6	40.2–487.5	94.7–275.7	95.3–191	164.1–205.2
Pb	average	24.7	21.9	19.0	39.4	35.2	29.0	38.6
	range	14.8–38.6	15.7–28.9	12.3–24.3	10.6–68.6	21.6–66.0	21318.0	31.5–46.4

**Table 4:** EF Average Values of PTEs in Bentonite Samples

PTEs	the average value of the enrichment factor							
	BQ1	BQ2	BQ3	BQ4	BQ5	BQ6	BQ7	all bentonite
Ti	1.34	1.76	0.86	1.14	0.82	0.45	0.16	1.03
V	1.71	2.15	1.47	1.47	1.26	0.41	0.23	1.33
Cr	3.13	5.00	1.44	0.94	1.02	0.17	0.03	2.08
Mn	1.40	1.25	0.48	0.79	0.54	1.24	0.23	0.97
Fe	1.11	1.36	0.82	0.91	0.76	0.44	0.19	0.86
Co	5.42	5.88	3.76	5.71	3.22	7.50	1.30	5.33
Ni	5.14	5.83	2.82	3.87	2.87	4.36	0.79	4.17
Cu	0.95	1.12	0.75	0.61	0.57	0.32	0.10	0.69
Zn	1.16	1.31	0.83	1.06	0.92	0.99	0.27	1.01
As	3.12	12.96	1.34	2.66	9.34	1.96	0.43	5.27
Zr	1.27	1.57	0.93	2.27	0.73	1.84	0.38	1.45
Pb	2.13	2.01	1.07	4.09	1.45	3.93	0.80	2.45

The concentrations of Ti in all samples varied from 491.8 to 7642.0 mg/kg with an average value of 3510.4 mg/kg. The average Ti concentration is lower than the earth’s crust average of 4500 mg/kg.^49^ The highest average concentration (HAC) value of Ti was analyzed in the bentonites from BQ2, while the lowest average concentration (LAC) value was in the bentonites from BQ6. According to the average Ti values, the BQs are ranked in descending order as follows: BQ2 > BQ1 > BQ5 > BQ3 > BQ4 > BQ7 > BQ6. From Table 4, The EF average values calculated for Ti in BQs varied from 0.2 to 1.8 with an average value of 1.0. All EF values indicate deficiency to minimal enrichment of Ti. The concentrations of V in all samples varied from 3.6 to 234.4 mg/kg with an average value of 94.7 mg/kg. The average V concentration is slightly higher than the earth’s crust average of 90 mg/kg.^49^ The HAC value of V was analyzed in the bentonites from BQ5, while the LAC value was in the bentonites from BQ6. According to the average V values, the BQs are ranked in descending order as follows: BQ5 > BQ3 > BQ2 > BQ1 > BQ4 > BQ7 > BQ6. The EF average values calculated for V in BQs varied from 0.2 to 2.1 with an average value of 1.3. The average EF value indicates deficiency to minimal enrichment of V. The concentrations of Cr in all samples varied from 2.7 to 537.1 mg/kg with an average value of 128.5 mg/kg. The average Cr concentration is higher than the earth’s crust average of 83 mg/kg.^49^ The HAC value of Cr was analyzed in the bentonites from BQ2, while the LAC value was in the bentonites from BQ7. According to the average Cr values, the BQs are ranked in the descending order as follows: BQ2 > BQ1 > BQ3 > BQ5 > BQ4 > BQ6 > BQ7. The EF average values calculated for Cr in BQs varied from 0.03 to 5.0 with an average value of 2.1. The average EF value indicates moderate enrichment of Cr. The concentrations of Mn in all samples varied from 120.4 to 2833.0 mg/kg with an average value of 740.8 mg/kg. The average Mn concentration is lower than the earth’s crust average of 1000 mg/kg.^49^ The HAC value of Mn was analyzed in the bentonites from BQ1, while the LAC value was in the bentonites from BQ4. According to the average Mn values, the BQs are ranked in descending order as follows: BQ1 > BQ2 > BQ7 > BQ6 > BQ5 > BQ3 > BQ4. The EF average values calculated for Mn in BQs varied from 0.2 to 1.4 with an average value of 1.0. The average EF value indicates deficiency to minimal enrichment of Mn. The concentrations of Fe in all samples varied from 3968.0 to 59310.0 mg/kg with an average value of 30568.98 mg/kg. The average Fe concentration is lower than the earth’s crust average of 46,500 mg/kg.^49^ The HAC value of Fe was analyzed in the bentonites from BQ2, while the LAC value was in the bentonites from BQ6. According to the average Fe values, the BQs are ranked in the descending order as follows: BQ2 > BQ1 > BQ5 > BQ3 > BQ4 > BQ7 > BQ6. The EF average values calculated for Fe in BQs varied from 0.2 to 1.4 with an average value of 0.9. The average EF value indicates deficiency to minimal enrichment of Fe. The concentrations of Co in all samples varied from 43.0 to 96.0 mg/kg with an average value of 66.9 mg/kg. The average Co concentration is approximately four times higher than the earth’s crust average of 18 mg/kg.^49^ The HAC value of Co was analyzed in the bentonites from BQ2, while the LAC value was in the bentonites from BQ6. According to the average Co values, the BQs are ranked in the descending order as follows: BQ2 > BQ1 > BQ7 > BQ3 > BQ5 > BQ4 > BQ6. The EF average values calculated for Co in BQs varied from 1.3 to 7.5 with an average value of 5.3. The average EF value indicates significant enrichment of Co. The concentrations of Ni in all samples varied from 79.5 to 355.1 mg/kg with an average value of 168.1 mg/kg. The average Ni concentration is approximately two times higher than the earth’s crust average of 58 mg/kg.^49^ The HAC value of Ni was analyzed in the bentonites from BQ2, while the LAC value was in the bentonites from BQ6. According to the average Ni values, the BQs are ranked in the descending order as follows: BQ2 > BQ1 > BQ5 > BQ3 > BQ7 > BQ4 > BQ6. The EF average values calculated for Ni in BQs varied from 0.8 to 5.8 with an average value of 4.2. The average EF value indicates moderate enrichment of Ni except for BQ1 and BQ2, which are significant enrichment of Ni. The concentrations of Cu in all samples varied from 3.3 to 58.7 mg/kg with an average value of 25.1 mg/kg. The average Cu concentration is approximately two times lower than the earth’s crust average of 47 mg/kg.^49^ The HAC value of Cu was analyzed in the bentonites from BQ2, while the LAC value was in the bentonites from BQ6. According to the average Cu values, the BQs are ranked in the descending order as follows: BQ2 > BQ3 > BQ1 > BQ5 > BQ4 > BQ7 > BQ6. From Table 4, the EF average values calculated for Cu in BQs varied from 0.1 to 1.1 with an average value of 0.7. All EF values indicate deficiency to minimal enrichment of Cu. The concentrations of Zn in all samples varied from 14.4 to 198.4 mg/kg with an average value of 61.8 mg/kg. The average Zn concentration is lower than the earth’s crust average of 83 mg/kg.^49^ The HAC value of Zn was analyzed in the bentonites from BQ5, while the LAC value was in the bentonites from BQ6. According to the average Zn values, the BQs are ranked in the descending order as follows: BQ5 > BQ1 > BQ2 > BQ7 > BQ3 > BQ4 > BQ6. From Table 4, the EF average values calculated for Zn in BQs varied from 0.3 to 1.3 with an average value of 1.0. All EF values indicate deficiency to minimal enrichment of Zn. The concentrations of As in all samples varied from <0.8 to 53.0 mg/kg with an average value of 9.3 mg/kg. The average As concentration is approximately five times higher than the earth’s crust average of 1.7 mg/kg.^49^ The HAC value of As was analyzed in the bentonites from BQ5, while the LAC value was in the bentonites from BQ6. According to the average As values, the BQs are ranked in the descending order as follows: BQ5 > BQ2 > BQ1 > BQ4 > BQ7 > BQ3 > BQ6. The EF average values calculated for As in BQs varied from 0.4 to 13.0 with an average value of 5.3. The average EF value indicates significant enrichment of As. The concentrations of Zr in all samples varied from 40.2 to 487.5 mg/kg with an average value of 172.5 mg/kg. The average Zr concentration is slightly higher than the earth’s crust average of 170 mg/kg.^49^ The HAC value of Zr was analyzed in the bentonites from BQ4, while the LAC value was in the bentonites from BQ6. According to the average Zr values, the BQs are ranked in the descending order as follows: BQ4 > BQ7 > BQ2 > BQ1 > BQ3 > BQ5 > BQ6. The EF average values calculated for Zr in BQs varied from 0.4 to 2.3 with an average value of 1.5. The average EF value indicates deficiency to minimal enrichment of Zr. The concentrations of Pb in all samples varied from 10.6 to 68.6 mg/kg with an average value of 27.9 mg/kg. The average Pb concentration is approximately two times higher than the earth’s crust average of 16 mg/kg.^49^ The HAC value of Pb was analyzed in the bentonites from BQ4, while the LAC value was in the bentonites from BQ3. According to the average Pb values, the BQs are ranked in the descending order as follows: BQ4 > BQ7 > BQ5 > BQ6 > BQ1 > BQ2 > BQ3. The EF average values calculated for Pb in BQs varied from 0.8 to 4.1 with an average value of 2.5. The average EF value indicates moderate enrichment of Pb.

## 4 Conclusions

In this study, the PTE (Ti, V, Cr, Mn, Fe, Co, Ni, Cu, Zn, As, Zr, and Pb) contents of bentonite samples produced in Turkey were investigated for the first time in detail. As a result of the study, it was revealed that the concentrations of Cr, Co, Ni, As, Zr, and Pb analyzed in bentonite samples were enriched according to the average concentrations in Earth’s crust. In addition, generally, the highest PTEs were analyzed in samples collected from quarry-coded BQ2, while the lowest PTEs were analyzed in quarry-coded BQ6. According to the average values of the EF, arsenic and cobalt are found significantly enriched in the investigated bentonite samples compared to an average of Earth’s crust.

The data obtained in this study are information that can raise awareness for both the end uses of bentonite and the workers in BQs. In addition, the distribution of PTEs in quarries may form a prospective database. To eliminate the situations that may threaten the health of the workers, it should be mandatory to take necessary measures such as preventing the workers from inhaling dust.

## References

[ref1] Khurshid C. A.; Mahdi K.; Ahmed O. I.; Osman R.; Rahman M.; Ritsema C. Assessment of Potentially Toxic Elements in the Urban Soil and Plants of Kirkuk City in Iraq. Sustainability 2022, 14, 5655 10.3390/su14095655.

[ref2] Tyagi N.; Upadhyay M. K.; Majumdar A.; Pathak S. K.; Giri B.; Jaiswal M. K.; Srivastava S. An assessment of various potentially toxic elements and associated health risks in agricultural soil along the middle Gangetic basin India. Chemosphere 2022, 300, 134433 10.1016/j.chemosphere.2022.134433. 35390408

[ref3] Liu P.; Zhang Y.; Feng N.; Zhu M.; Tian J. Potentially toxic element (PTE) levels in maize, soil, and irrigation water and health risks through maize consumption in northern Ningxia China. BMC Public Health 2020, 20, 1729 10.1186/s12889-020-09845-5. 33198713 PMC7670719

[ref4] Marín J.; Colina M.; Ledo H.; Gardiner P. H. E. Ecological risk by potentially toxic elements in surface sediments of the Lake Maracaibo (Venezuela). Environ. Eng. Res. 2022, 27, 210232 10.4491/eer.2021.232.

[ref5] Pavlović P.; Sawidis T.; Breuste J.; Kostić O.; čakmak D.; Đorđević D.; Pavlović D.; Pavlović M.; Perović V.; Mitrović M. Fractionation of potentially toxic elements (PTEs) in urban soils from Salzburg, Thessaloniki and Belgrade: An insight into source identification and human health risk assessment. Int. J. Environ. Res. Public Health 2021, 18, 6014 10.3390/ijerph18116014. 34205068 PMC8199883

[ref6] Santoyo-Martínez M.; Aguilera A.; Gallegos Á.; Puente C.; Goguitchaichvili A.; Bautista F. Pollution Levels and Potential Health Risks of Potentially Toxic Elements in Indoor and Outdoor Dust during the COVID-19 Era in Gómez Palacios City Mexico. Land 2023, 12, 29 10.3390/land12010029.

[ref7] Turhan Ş.; Metin O.; Hançerlioğulları A.; Kurnaz A.; Duran C. Determination of elemental concentrations of radionuclides in Turkish bentonite and calculation of radiogenic heat generation. Int. J. Environ. Anal. Chem. 2022, 2140419 10.1080/03067319.2022.2140419.

[ref8] Abdul-Wahab S. A.; Marikar F. A. The environmental impact of gold mines: pollution by heavy metals. Cent. Eur. J. Eng. 2012, 2, 304–313. 10.2478/s13531-011-0052-3.

[ref9] Wongsasuluk P.; Tun A. Z.; Chotpantarat S.; Siriwong W. Related health risk assessment of exposure to arsenic and some heavy metals in gold mines in Banmauk Township, Myanmar. Sci. Rep. 2021, 11, 22843 10.1038/s41598-021-02171-9. 34819590 PMC8613182

[ref10] Mandal M.; Bhattacharya S.; Paul S. Assessing the level of contamination of metals in surface soils at thermal power area: Evidence from developing country (India). Environ. Chem. Ecotoxicol. 2022, 4, 37–49. 10.1016/j.enceco.2021.11.003.

[ref11] Karn R.; Ojha N.; Abbas S.; Bhugra S. A review on heavy metal contamination at mining sites and remedial techniques. IOP Conf. Ser.: Earth Environ. Sci. 2021, 796, 012013 10.1088/1755-1315/796/1/012013.

[ref12] Zhao G.; Li X.; Zhu J.; Zhao X.; Zhang J.; Zhai J. Pollution assessment of potentially toxic elements (PTEs) in soils around the Yanzhuang Gold Mine tailings pond, Pinggu County, Beijing, China. Int. J. Environ. Res. Public Health 2021, 18, 7240 10.3390/ijerph18147240. 34299689 PMC8308061

[ref13] Yu Y.; Liu W.; Luo H.; He L.; Liu H.; Xu R.; Zhang L.; Wang Y.; Wu G.; Wei F. Assessing the Risk of Total and Available Potentially Toxic Elements in Agricultural Soil in Typical Mining Areas in Xiangjiang River Basin Hunan Province. Minerals 2021, 11, 953 10.3390/min11090953.

[ref14] Kurnaz A.; Turhan Ş.; Metin O.; Altıkulaç A.; Duran C. Evaluation of terrestrial radionuclide levels and concomitant radiological risks of bentonites used in many industries. Int. J. Environ. Health Res. 2022, 2022, 2120190 10.1080/09603123.2022.2120190. 36062411

[ref15] Babahoum N.; Ould H. M. Characterization and purification of Algerian natural bentonite for pharmaceutical and cosmetic applications. BMC Chem. 2021, 15, 50 10.1186/s13065-021-00776-9. 34470665 PMC8411512

[ref16] Kutlić A.; Bedeković G.; Sobota I. Bentonite processing. Rud. Geol. Naft. Zb. 2012, 24, 61–65.

[ref17] Ozguven F.; Pekdemir A.; Onal M.; Sarıkaya Y. Characterization of a bentonite and its permanent aqueous suspension. J. Turk. Chem. Soc., Sect. A 2020, 7, 11–18. 10.18596/jotcsa.535937.

[ref18] Ferreira J. F.; Costa F. P. D.; Borborema L. F. D.; Arimateia R. R. D.; Leite R. S.; Apolinário R. C.; Pinto H. C.; Rodrigues A. M.; Neves G. D. A.; Menezes R. R. Incorporation of Bentonite Mining Waste in Ceramic Formulations for the Manufacturing of Porcelain Stoneware. Sustainability 2022, 14, 15973 10.3390/su142315973.

[ref19] Araújo M. E. B.; Silva V. C.; Fernandes J. V.; Cartaxo J. M.; Rodrigues A. M.; Menezes R. R.; de Araújo Neves G. Innovative adsorbents based on bentonite mining waste for removal of cationic dyes from wastewater. Environ. Sci. Pollut. Res. 2022, 29, 90446–90462. 10.1007/s11356-022-22083-z. 35871192

[ref20] Liu Z.; Zhou S. Adsorption of copper and nickel on Na-bentonite. Process Saf. Environ. Prot. 2010, 88, 62–66. 10.1016/j.psep.2009.09.001.

[ref21] Chaves L. H. G.; Tito G. A. Cadmium and copper adsorption on bentonite: effects of pH and particle size. Rev. Ciênc. Agron. 2011, 42, 278–284. 10.1590/S1806-66902011000200004.

[ref22] Turan N.; Ozgonenel O. Study of montmorillonite clay for the removal of copper (II) by adsorption: full factorial design approach and cascade forward neural network. Sci. World J. 2013, 342628 10.1155/2013/342628. PMC3881663 24453833

[ref23] Malamis S.; Katsou E. A review on zinc and nickel adsorption on natural and modified zeolite, bentonite and vermiculite: examination of process parameters, kinetics and isotherms. J. Hazard. Mater. 2013, 252–253, 428–461. 10.1016/j.jhazmat.2013.03.024. 23644019

[ref24] Dutta J.; Mishra A. K. Influence of the presence of heavy metals on the behavior of bentonites. Environ. Earth Sci. 2016, 75, 993 10.1007/s12665-016-5811-2.

[ref25] Tahervand S.; Jalali M. Sorption and desorption of potentially toxic metals (Cd, Cu, Ni and Zn) by soil amended with bentonite, calcite and zeolite as a function of pH. J. Geochem. Explor. 2017, 181, 148–159. 10.1016/j.gexplo.2017.07.005.

[ref26] Tohdee K.; Asadullah L. K. Enhancement of adsorption efficiency of heavy metal Cu(II) and Zn(II) onto cationic surfactant modified bentonite. J. Environ. Chem. Eng. 2018, 6, 2821–2828. 10.1016/j.jece.2018.04.030.

[ref27] Nartowska E. The effects of potentially toxic metals (copper and zinc) on selected physical and physico-chemical properties of bentonites. Heliyon 2019, 5, e02563 10.1016/j.heliyon.2019.e02563. 31667404 PMC6812193

[ref28] Kakaei S.; Khameneh E. S.; Rezazadeh F.; Hosseini M. H. Heavy metal removing by modified bentonite and study of catalytic activity. J. Mol. Struct. 2020, 1199, 126989 10.1016/j.molstruc.2019.126989.

[ref29] Altun T. Preparation and application of glutaraldehyde cross-linked chitosan coated bentonite clay capsules: Chromium (VI) removal from aqueous solution. J. Chil. Chem. Soc. 2020, 65, 4790–4797. 10.4067/S0717-97072020000204790.

[ref30] Ahmed A. M.; Ayad M. I.; Eledkawy M. A.; Darweesh M. A.; Elmelegy E. M. Removal of iron, zinc, and nickel-ions using nano bentonite and its applications on power station wastewater. Heliyon 2021, 7, e06315 10.1016/j.heliyon.2021.e06315. 33681500 PMC7930288

[ref31] AlTowyan L.; AlSagabi S.; AlAjyan T.; AlSulami K.; Goumri-Said S. The removal of manganese ions from industrial wastewater using local Saudi and commercial bentonite clays. Groundwater Sustainable Dev. 2022, 19, 100821 10.1016/j.gsd.2022.100821.

[ref32] Ece D.; Aydemir Ö. E.; Özkutlu F. The effect of Ca-bentonite application on cadmium uptake and shoot dry matter of bread wheat. Turk. J. Nat. Sci. 2022, 11, 50–54. 10.46810/tdfd.1120664.

[ref33] Mutar R. F.; Saleh M. A. Optimization of arsenic ions adsorption and removal from hospitals wastewater by nano-bentonite using central composite design. Mater. Today: Proc. 2022, 60, 1248–1256. 10.1016/j.matpr.2021.08.213.

[ref34] Altıkulaç A.; Turhan Ş.; Kurnaz A.; Gören E.; Duran C.; Hançerlioğulları A.; Uğur F. A. Assessment of the enrichment of heavy metals in coal and its combustion residues. ACS Omega 2022, 7, 21239–21245. 10.1021/acsomega.2c02308. 35935287 PMC9347966

[ref35] Ene A.; Sion A. B.; Lucian G. Determination of heavy metals in soils using XRF technique. Rom. J. Phys. 2010, 55, 815–820.

[ref36] Oyedotun T. D. T. X-ray fluorescence (XRF) in the investigation of the composition of earth materials: a review and an overview. Geol. Ecol. Landscapes 2018, 2, 148–154. 10.1080/24749508.2018.1452459.

[ref37] Turhan Ş.; Garad A. M. K.; Hançerlioğulları A.; Kurnaz A.; Gören E.; Duran C.; Karataşlı M.; Altıkulaç A.; Savacı G.; Aydın A. Ecological assessment of heavy metals in soil around a coal–fired thermal power plant in Turkey. Environ. Earth Sci. 2020, 79, 134 10.1007/s12665-020-8864-1.

[ref38] Povarov V. G.; Kopylova T. N.; Sinyakova M. A.; Rudko V. A. Quantitative determination of trace heavy metals and selected rock-forming elements in porous carbon materials by the X-ray fluorescence method. ACS Omega 2021, 6, 24595–24601. 10.1021/acsomega.1c03217. 34604641 PMC8482509

[ref39] Panebianco S.; Mazzoleni P.; Barone G.; Musumarra A.; Pellegriti M. G.; Pulvirenti A.; Scordino A.; Cirvilleri G. Feasibility study of tomato fruit characterization by fast XRF analysis for quality assessment and food traceability. Food Chem. 2022, 383, 132364 10.1016/j.foodchem.2022.132364. 35193091

[ref40] Altıkulaç A. Investigation of radiological and chemical contents of bauxite ore extracted in Turkey. ACS Omega 2022, 7, 39917–39923. 10.1021/acsomega.2c04203. 36385831 PMC9648063

[ref41] Krista E. M. A.; Turhan Ş.; Kurnaz A.; Hançerlioğulları A. Determination of the elemental composition of mica samples from Turkey. J. Polytech. 2022, 25, 1271–1279. 10.2339/politeknik.1056220.

[ref42] Rosa J.; de Carvalho L. A. E. B.; Ferreira M. T.; Gonçalves D.; Marques M. P. M.; Gil F. P. S. C. Chemical trace XRF analysis to detect sharp force trauma in fresh and burned bone. Sci. Justice 2022, 62, 484–493. 10.1016/j.scijus.2022.07.007. 36336441

[ref43] Turhan Ş.; Tokat S.; Kurnaz A.; Altıkulaç A. Distribution of elemental compositions of zeolite quarries and calculation of radiogenic heat generation. Int. J. Environ. Anal. Chem. 2022, 109, 7851–7862. 10.1080/03067319.2020.1839439.

[ref44] Qingyu L.; Zhi L.; Womack A.; Tian J.; Wang F. A preliminary analysis of pottery from the Dantu site, Shandong, China: Perspectives from petrography and WD-XRF. J. Archaeol. Sci. Rep. 2023, 47, 103710 10.1016/j.jasrep.2022.103710.

[ref45] Al-Kahtany K.; El-Sorogy A. S. Contamination and health risk assessment of surface sediments along Ras Abu Ali Island, Saudi Arabia. J. King Saud Univ. Sci. 2023, 35, 102509 10.1016/j.jksus.2022.102509.

[ref46] Poh S. C.; Tahi N. M. The common pitfall of using enrichment factor in assessing soil heavy metal pollution. Malays. J. Anal. Sci. 2017, 21, 52–59.

[ref47] Turhan Ş. Evaluation of agricultural soil radiotoxic element pollution around a lignite-burning thermal power plant. Radiochim. Acta 2020, 108, 77–85. 10.1515/ract-2018-3051.

[ref48] Krishna A. K.; Govil P. K. Assessment of heavy metal contamination in soils around Manali industrial area, Chennai Southern India. Environ. Geol. 2008, 54, 1465–1472. 10.1007/s00254-007-0927-z.

[ref49] Yaroshevsky A. A. Abundances of chemical elements in the Earth’s crust. Geochem. Int. 2006, 44, 48–55. 10.1134/S001670290601006X.

